# O.5.1-9 Doping intention in cyclists: interaction effects of achievement goals and athlete burnout

**DOI:** 10.1093/eurpub/ckad133.239

**Published:** 2023-09-11

**Authors:** Karine Corrion, Valentine Filleul, Emma Vivier, Eric Meinadier, Jacky Maillot, Sandrine Isoard-gautheur, Eric Fruchart, Fabienne d’Arripe-Longueville

**Affiliations:** Université Côte-d’Azur LAMHESS, Nice, France; Université Côte-d’Azur LAMHESS, Nice, France; Université Côte-d’Azur LAMHESS, Nice, France; French Federation of Cycling; French Federation of Cycling; University of Grenoble Alpes, SENS, France; University of Perpignan, LEPSA, France; karine.corrion@univ-cotedazur.fr; Université Côte-d’Azur LAMHESS, Nice, France

## Abstract

**Purpose:**

Doping remains a significant issue in cycling, leading to potential health, economic or mediatic consequences. The role of sociocognitive variables in doping is well identified and the 2 x 2 model of the achievement goals theory suggests that mastery-approach (MAp) goals are protective whereas mastery-avoidance (MAv), performance (approach PAp, avoidance PAv) goals are maladaptive. Previous research has shown that burnout would be related to doping in academic and professional contexts (Patel, 2019) and more recently in sport (Filleul et al., 2022). However, the specific roles of achievement goals and burnout regarding doping remain to be identified. We expected to observe an interaction effect between achievement goals and burnout on doping intention.

**Methods:**

Our study applied Anderson’s methodology (2008) with realistic scenarios in which the cyclist was faced with the decision to use doping substances (e.g., Fruchart et al., 2019). We manipulated achievement goals (x4; Riou et al., 2012) and athlete burnout (x2; Isoard-Gautheur et al., 2018; Shirom & Melamed, 2006) yielding to eight scenarios. The participants were 71 French competitive road cyclists (58 males, Mage = 17.24, *SD* = 3.34) recruited in cycling structures. We assessed participants’ both explicit and implicit doping attitudes at baseline and then measured the doping intention for each scenario, on a 11-point Likert-scale. Repeated Measures ANOVAs were used to examine the within-subject interaction effects of the manipulated variables on doping intention. Post hoc tests were then conducted for comparisons between conditions.

**Results:**

We found a significant interaction effect between achievement goals and athlete burnout on doping intention (F(2.485, 173.924) = 4.506, *p* = .008, *η*_p_^2^ = .060). Cyclists reported significantly higher doping intention (*P*_holm_ <.01) in the scenarios with: (a) PAp-burnout rather than in MAp-burnout, (b) PAp-burnout rather than in PAp-no burnout, and (c) MAv-burnout rather than in MAv-no burnout situation.

**Conclusions:**

Our findings suggest that burnout may play a key role in understanding the effects of achievement goals on doping intentions. We recommend sport organizations and coaches to focus on promoting mastery-approach climates and on implementing interventions to prevent burnout, such as stress management and coping strategies.

